# Relationship between Daily Physical Activity During Last Month of Pregnancy and Pregnancy Outcome

**Published:** 2011-01-01

**Authors:** M Koushkie Jahromi, B Namavar Jahromi, S Hojjati

**Affiliations:** 1 Department of Physical and Sport Sciences, Shiraz University, Shiraz, Iran; 2 Department of Obstetric and Gynecology, Shiraz University of Medical Sciences, Shiraz, Iran

**Keywords:** Physical activity, Exercise, Pregnancy, Outcome

## Abstract

**Background:**

Previous researchers have evaluated the influence of physical exercise or physical activity on pregnancy outcome, but the influence of daily physical activities in details including energy expenditure, biomechanical load and exercise before and during pregnancy have remained unclear. This study evaluates the relationship between daily physical activities as a biomechanical load and energy expenditure and physical exercise during household activities with birth weight, type of delivery and Apgar score.

**Methods:**

The participants of this study were household, first parity women who referred to a prenatal care center in southern Iran. 132 volunteer women were eligible to be enrolled according to their general health and not having any absolute or relative limitation for participating in any kind of activity. Information about daily physical activity was collected through a personal interview using a structured questionnaire during two separate days of ninth month of pregnancy. Data on delivery were recorded from recorded documents of mothers in the hospital.

**Results:**

There was no relationship between biomechanical and energy load and birth weight. There was a significant correlation between Apgar score and biomechanical and energy load. Infants of mothers who exercised before or during pregnancy had a significant higher weight than the non-exercise group. Apgar score indicated no significant difference among those having exercise and those without before and during pregnancy. There was no significant difference in the biomechanical load and energy expenditure in the two types of delivery.

**Conclusion:**

Daily activities in normal range do not induce any harmful effect on birth weight; increasing biomechanical load as a result of some maternal body postures that may be harmful for infant health at birth time. Physical exercise before and during pregnancy may have a positive effect on birth weight.

## Introduction

Until a few years ago, many women were recommended to reduce daily activities or stop occupational works during their final months of pregnancy.[1] However, recently physical activity during pregnancy is recommended and many studies have indicated the positive influence of exercise especially light to moderate types (exercise intensity: 40-55% vo2max, duration: 20-45 , moderate: walking or swimming, frequency: 2-3 days per week) during the third trimester of pregnancy and encourage women for participation in physical activity with no contradictions.[2] It has been discussed that physical activity during pregnancy may influence maternal body thermal response and shift blood concentration from uterus and placenta to extremities. Although, many available researches indicated that moderate level of physical exercise during pregnancy is safe for mothers and fetuses,[2] physical activity during pregnancy still raises some controversies. Daily physical activity can include occupational and exercise activities which may influence on pregnancy outcome including birth weight, type of delivery and Apgar score (health condition of the child at birth which is evaluated by physicians or midwives).

Birth weight is an important factor which influence on general health and chances of survival of infants. Low birth weight (LBW) has a great risk of mortality and long term morbidity. Recent studies indicated that LBW is associated with increased risk of metabolic disorders and cardiovascular diseases.[3] Established risk factors for preterm birth are previous LBW or preterm delivery, gestational bleeding and cervical and uterine anomalies. Some probable risk factors are urogenital infections, maternal age and weight gain, parity, and dietary intake. Daily or leisure time physical activities can be risk factors for LBW but with insufficient related data.[1] Until a few decades ago, pregnant women were recommended to reduce their activities and even stop their occupational work especially during the final months of pregnancy.[4] Some studies have measured the influence of different occupational physical activities on birth weight and reported no significant effect on birth weight,[5] and some studies have indicated that job related physical activity is related to preterm delivery and LBW.[5][6][7][8] Many data from some studies indicate the positive effects of exercise on birth weight[9] while some findings showed no significant effect of exercise on birth weight,[10] and few studies noticed negative effects.[11] In a study about influence of occupational physical activity on pregnancy duration and birth weight, it was shown that working for more than 24 hours a week and standing for more than six hours a day were associated with LBW.[7]

There are controversies on leisure time exercise habits and pregnancy outcome. Some researches demonstrated that exercise during pregnancy may influence positively on maternal outcome.[10] Most studies found no effect for exercise on labor.[12] Some studies revealed that exercise during pregnancy can reduce the risk of cesarean section.[13] Sibley et al. found that 1st min Apgar score is higher in women who swam during pregnancy than the control group.[14] They noticed that demanding physical activities did not have a harmful effect on birth outcome such as birth weight, gestational age at delivery, preterm birth and survival.[15] No study was available related to maternal daily activity consisting biomechanical load and energy expenditure and Apgar score. Different study designs, exercise regime, maternal posture and energy balance may cause so much variety in results. It is possible that occupational physical activity including reaching, bending, and lifting may increase the pressure on the spine and so to increase the abdominal pressure and may influence on pregnancy outcome.[16] One of the limitations in most performed studies is that they investigated daily physical activity in employed women or only to exercising women while in many countries several women may be households without any occupation. According to one general belief that physical activity during the last month of pregnancy is useful for delivery outcome, this study was undertaken to evaluate relationship between biomechanical load and energy expenditure (estimating according to all daily physical activities during the last month of pregnancy) and physical exercise (before and during pregnancy) with pregnancy outcome in household women.

## Materials and Methods

The Study subjects were drawn from pregnant women who received prenatal care in the Fars Province, southern Iran from April 2007 to April 2008. Being household, first parity, 33-35 weeks of pregnancy and singleton babies were inclusion criteria. The women were invited after informing them of the aims of study and with a voluntarily participation were enrolled. The exclusion criteria were previous cesarean section, diabetes, hypertension, heart diseases, chronic renal diseases, RH sensitization, current use of corticosteroids, cervical incompetence, receipt of antibiotics within the two weeks preceding the interview, previous treatments with tocolytic agents between the beginning of pregnancy and the date of interview, abnormal weight gain for gestational age (base on woman's body mass index=BMI: weight/height2)[17] and any kind of bleeding during pregnancy. 142 women at 33-35 weeks of gestation were interviewed and our actual participants were 132 women (10 women did not refer to the hospital for delivery).

The participants were interviewed using a structured questionnaire. Information on the current daily activity of a usual day and weekend were obtained by recording all activities during 24 hours in a chronological order. Items of questions were time spent on activities in different postures such as sitting, standing, lying, sleeping, walking alternate with standing, bending and doing regular physical exercise in the form of walking or aerobics at least twice a week and more than half an hour. In addition, information on physical exercise before pregnancy was included. Questions on reproductive history, level of education of participants and their husbands, husband outcome, and maternal weight before pregnancy were provided. They were asked to report any change in their daily activity from the beginning of last month until delivery. Birth weight was recorded immediately after delivery and Apgar score during the 1st minute of delivery. Maternal weight gain during pregnancy and other factors which may influence birth weight including education, gestational hypertension, preeclampsia, prenatal death and duration of pregnancy based on the date of last menstruation and ultrasonography before the 20th week of pregnancy were collected according to maternal document in prenatal care center. Maternal energy intake was controlled using dietary recommendation, which was according to maternal weight gain chart during pregnancy.

Energy expenditure was assigned to different activities according to the mean time devoted to different activities during the last month multiplied by weight. These weight estimated the rate at which basal metabolism increased for a special activity for adult women with normal body weight: sitting (1.5), standing (2.0), walking (4.0), standing alternate with walking (2.5) and bending (4.0).[18]

Biomechanical load was assigned according to the pressure on spinal cord during different body postures, which were associated with intra-abdominal pressure. Daily biomechanical load was calculated by sum of mean hours devoted to a special activity during the last month of pregnancy multiplied by the weight in the following parenthesis which indicate the rate at which intra-abdominal pressure increased during a given posture (walking is as the reference): sitting (3.0), standing (2.0), walking (1), standing alternate with walking (1.5) and bending (8.0).[19] All subjects voluntarily participated in this study and fulfilled informed consent form. They were assured of privacy of their responses.

Relationship between birth weight with variables including energy expenditure, biomechanical load and duration of every maternal posture was analyzed using Pearson correlation test. Comparison of birth weight and Apgar scores between infants of mothers who participated in physical exercise before or during pregnancy, and biomechanical and energy load in two types of deliveries were evaluated using independent t test. MCNemar test was used to compare the frequency of two types of deliveries in exercise group before and during pregnancy and those without any exercise.

## Results

Table 1 presents the characteristics of 132 healthy women participated in this study. All women were from middle or high socioeconomic class. All received a balanced diet containing adequate calories (according to maternal weight gain during pregnancy).

**Table 1:** Main characteristics of participants a

**Parameters**	**All live births (N= 132)**	**Exercisers before pregnancy (N=76)**	**Non- exercisers before pregnancy (N=56)**	**Exercisers during pregnancy (N=70)**	**Non exercisers during pregnancy (N=62)**
Age (years)	23±4.7	22±5.1	23±3.91	23±5.22	23±4.80
Gestational duration (LMP in weeks)	38.78±3.90	38.82±2.75	37.46±4.20	39.15±1.30	38.23±2.01
Educational level Up to middle school	11 (8.30%)	6 (7.89%)	5 (8.92%)	6 (8.57%)	5 (8.06%)
High school and diploma	82 (62.12%)	45 (59.21%)	36 (64.28%)	43 (61.42%)	40 (64.51%)
University degree and above	39 (29.54%)	25 (32.89%)	15 (26.78%)	21 (30%)	17 (27.41%)
Height (cm)	162±6.61	162.42±6.92	163.10±6.32	163.13±6.62	162.13±6.73
Pre pregnancy Weight (kg)	61.23±10.61	61.71±10.33	60.39±11.39	63.51±12.40	59.45±9.17
Pre pregnancy BMI (kg/m2)	23.22±4	23.53±4.37	22.64±3.74	22.42±3.58^b^	24.19±4.65^b^
maternal weight gain (kg)	14.43±3.29	13.51±4.16	13.85±5.31	13.78±4.25	14.12±2.18
Infant sex (%)					
Boy	55.9%	58.1%	53.7%	54.2%	57.6%
girl	44.1%	41.9%	46.3%	45.8%	42.4%
Type of delivery					
Vaginal	56%	29.41%	19.11%	29.85%	20.84%
Cesarean	76%	29.41%	22.05	26.85%	22.05%
Birth weight (kg)	3.06±0.58	3.21±0.53	2.88±0.60	3.23±0.40	2.91±0.36

^a^ LMP= according to last menstrual period, cm=centimeter, kg=kilogram,

^b^ significant difference between BMI of exercisers and non exercisers during pregnancy (p<0.05)

A negative significant correlation was visible between biomechanical load and Apgar score (r=0.25, p=0.050), but without any significant relationship between other variables including birth weight and biomechanical load (r=0.06, p=0.678), birth weight and energy expenditure (r=0.10, p=478) and Apgar score and energy expenditure (r=-0.23, p=109) during pregnancy (Table 2). There were no significant differences in type of delivery in the two groups of mothers who had exercise before (p=0.291) and during pregnancy (p=0.594) (Table 3). There were significant differences in birth weight of infants of mothers who exercised and did not exercise during pregnancy (p=0.021) and before pregnancy (p=0.021). Apgar score did not indicate to any significant difference in mothers with exercise and those without any exercise before (p=0.321) and during pregnancy (p=0.821). There were not significant differences in biomechanical load (p=0.165) and energy expenditure (p=0.309) in the two types of deliveries (Table 4)

**Table 2:** Correlation of biomechanical and energy load with birth weight and Apgar score using Pearson correlation test

		**Biomechanical load**	**Energy load**
Birth weight	Correlation coefficient	0.06	0.106
	*p*	0.678	0.478
	Number	123	123
Apgar score	Correlation coefficient	-0.25	-0.23
	*p*	0.050^a^	0.109
	Number	123	123

^a^ Significant correlation between Apgar score and biomechanical load

**Table 3:** Comparison of types of delivery in exercise and non-exercise group before and during pregnancy using MC Nemar test

**Parameters**	**Exercisers before pregnancy (N=76)**	**Non exercisers before pregnancy (N=56)**	**Exercisers during pregnancy (70)**	**Non exercisers during pregnancy (62)**
Type of delivery				
Vaginal	53 (69.73%)	40 (71.42%)	50 (71.42%)	43 (69.35%)
Cesarean	23 (30.26%)	16 (28.57%)	20 (28.71%)	19 (30.64%)
*P*	0.291		0.594	

**Table 4:** Comparison of some study variables between groups using t test

	**Number**	**Mean±SD**	**P**
Birth weight (kg)			
Pre pregnancy Exercise	76	3.21±0. 53	0.021^a^
Pre pregnancy Non exercise	54	2.88±0.60	
Birth weight (kg)			
Exercise During pregnancy	70	3.23±0.40	0.021^a^
Non exercise During pregnancy	58	2.91±0.36	
Biomechanical load			
Cesarean	39	36.51± 8.88	0.165
Vaginal	93	32.72± 9.79	
Energy load			
Cesarean	39	28.47±5.82	0.309
Vaginal	93	26.77±5.61	
Apgar score (1 min)			
Pre pregnancy exercise	76	9.30±0. 34	0.321
Pre pregnancy non exercise	56	9.11±1.1	
Apar score (1 min)			
Exercise during pregnancy	70	9.2±0.68	0.823
Non exercise during pregnancy	62	9.17±1.01	

^a^ There are significant differences between birth weight in exercisers before and during pregnancy and non exercisers SD: standard deviation

## Discussion

The present study evaluated the influence of biomechanical load and energy expenditure on birth weight among primiparous, household Iranian women. Our findings indicated that energy expenditure rate was not significantly correlated with birth weight. All participants were healthy, primiparous women from a similar society (identical socioeconomic status) and all were household women with similar daily physical activity.

Results of the study indicated that maternal participation in exercise training before and during pregnancy was positively effective on birth weight (Table 4). Some other studies are in consistent with our results[20] while some others reported that light to moderate physical exercise during pregnancy would not increase the risk of low birth weight and did not indicate to any effect.[20][21][22][23] One limitation of this study is that 97% of mothers who exercised before pregnancy continued their exercise through the entire pregnancy period and we could not divide them to mothers with exercise during different stages of pregnancy which can affect our results. Maternal exercise in this study included brisk walking (80%) or aerobic exercises (20%) with moderate intensity (40-55% maximal heart rate), at least twice a week for 30 minute or more during the 9th month of pregnancy and any kind of exercise such as basketball, volleyball or track and field before pregnancy. According to a review, although physical exercise may cause reducing uteroplacental blood flow, hyperthermia, shortage of substrate (blood glucose) availability, and release of catecholamines which may induce uterine contractions during pregnancy, there were compensatory mechanisms toh protect the fetus.[12] Concern for fetal well being during exercise and during pregnancy are related to adequate blood flow to supply fetal oxygen requirements and adequate glucose and other substrates for the fetus. Researches indicate that pregnancy and exercise are complementary to each other, as exercise increases fat for energy needs which can cause increasing availability of glucose and oxygen for the fetus.[24]

Findings of this study showed no significant difference in the type of delivery in both groups of mothers who exercised before and during pregnancy and those without any exercise. Biomechanical load and energy expenditure did not show any significant difference in two types of deliveries. We can conclude that daily physical exercise and activity were not correlated with the type of delivery. Some studies found that exercise during pregnancy is positively related to the reduction in risk of cesarean section.[13] Most studies did not demonstrate any correlation between type of delivery and exercise.[12] This may indicate to a moderate difference in daily activity or light exercise in the last month of pregnancy that may not influence on the type of delivery. Socioeconomic factors are important in determining the activity level during pregnancy.[5] Population of this study consisted of about 98% households and 2% outside home workers and for a better study, we excluded outside home employed women as all participants were household and probably from a similar socioeconomic status. It probably may affect their daily activity and our results, so future research on participants of more extended region with different daily activities including urban and rural women, or employed and non-employed women with a more vigorous exercise may clarify the matter.

The strength of this study was evaluating daily activities as energy expenditure and biomechanical load (because total energy expenditure is very important in health) while in previous studies daily physical activity was evaluated as duration of some especial activities (e.g. standing, walking and sleeping). In addition to daily activities, physical exercise before and during pregnancy was also evaluated. Because accurate measurement of the component of physical activity can aid in our understanding of the dose response of physical activity and pregnancy outcome,[25] Combining the records of daily physical activity with interviews (for checking its accuracy) the validity of results increased.

The present study indicated that routine daily household activities during the last month of pregnancy was not associated with birth weight and moderate physical exercise during and before pregnancy is an important positive effective factor on birth weight. So we can suggest that daily activities in normal range are not harmful for infants and participating in moderate exercise before pregnancy and continuing it during pregnancy is useful for infant health. Daily activities in normal range do not induce any dangerous effect on birth weight, but increasing biomechanical load as a result of some maternal body postures may be hazardous for general health of infant at birth time.
